# Follow-Up Study of Effectiveness of 23-Valent Pneumococcal Polysaccharide Vaccine Against All-Type and Serotype-Specific Invasive Pneumococcal Disease, Denmark

**DOI:** 10.3201/eid3006.230975

**Published:** 2024-06

**Authors:** Katrine Finderup Nielsen, Lise Birk Nielsen, Tine Dalby, Frederikke Kristensen Lomholt, Hans-Christian Slotved, Kurt Fuursted, Zitta Barrella Harboe, Charlotte Sværke Jørgensen, Palle Valentiner-Branth

**Affiliations:** Statens Serum Institut, Copenhagen, Denmark (K. Finderup Nielsen, L.B. Nielsen, T. Dalby, F.K. Lomholt, H.-C. Slotved, K. Fuursted, Z.B. Harboe, C.S. Jørgensen, P. Valentiner-Branth);; Copenhagen University Hospital, North Zealand, Copenhagen (Z.B. Harboe);; University of Copenhagen, Copenhagen (Z.B. Harboe)

**Keywords:** invasive pneumococcal disease, 23-valent pneumococcal polysaccharide vaccine, *Streptococcus pneumoniae*, vaccine effectiveness, epidemiology, public health programs, pneumococcal-conjugated vaccines, pneumonia, bacteria, meningitis/encephalitis, respiratory infections, streptococci, Denmark

## Abstract

As a follow-up to a previous study, we investigated vaccine effectiveness (VE) of 23-valent pneumococcal polysaccharide vaccine (PPSV23) against invasive pneumococcal disease (IPD) among 1,254,498 persons >65 years of age as part of a vaccination program in Denmark during April 2020–January 2023. We assessed VE by using a Cox regression model and adjusted for age, sex, and underlying conditions. Using nationwide data, we estimated a VE of PPSV23 against all-type IPD of 32% and against PPSV23-serotype IPD of 41%. Because this follow-up study had more statistical power than the original study, we also estimated VE against IPD caused by PPSV23-serotypes excluding serotype 3; serotype 3; serotype 8; serotype 22F; PPSV23 non-PCV15 serotypes; PPSV23 non-PCV20 serotypes; and IPD over time. Our findings suggest PPSV23 vaccination can protect persons >65 years of age against IPD caused by all serotypes or serotype groupings, except serotype 3.

Invasive *Streptococcus pneumoniae* infections can cause deadly diseases such as meningitis and bacteremia. In Denmark, children <2 years of age have been routinely vaccinated against pneumococcal disease with pneumococcal conjugate vaccine (PCV): a 7-valent PCV (PCV7) since October 2007 and a 13-valent PCV (PCV13) since 2010 (*1*,*2*). For adults, pneumoccocal vaccination has only been recommended for those at increased risk for pneumococcal disease; whereas childhood vaccinations were free, adults had to pay. After PCV vaccines were added to the childhood vaccination program, the rate of invasive pneumococcal disease (IPD) declined drastically in children <2 years of age, and a simultaneus but less pronounced decline was detected in those >65 years of age due to herd immunity (*1*). The serotype distribution also changed in the oldest age group; whereas non-PCV13 serotypes accounted for 30% of IPD cases before the introduction of PCV in the childhood vaccination program, they accounted for 59% during 2011–2013 (*1*).

During the COVID-19 pandemic, there was a focus on preventing hospitalizations in those >65 years of age, who are at increased risk for both IPD (*3*,*4*) and severe COVID-19 (*5*,*6*). Therefore, the government of Denmark initiated a vaccination program to prevent IPD, using the 23-valent pneumococcal polysaccharide vaccine (PPSV23), aimed at persons >65 years of age (*7*). The program started on April 22, 2020, and was first aimed at persons >65 years of age who had an increased risk for IPD (e.g., those with chronic lung or heart disease). On June 15, 2020, the program was expanded to include all persons >65 years of age and ran until January 15, 2023. During the first year of the program, 61% of Denmark residents >65 years of age were vaccinated with PPSV23 (*8*). The most common IPD-causing serotypes in persons >65 years of age in Denmark during the study period were 3 (19%), 8 (12%) and 22F (7%). All 3 serotypes are included in PPSV23. PPSV23 has previously been shown to have an effect against IPD caused by the included serotypes in persons >60 years of age, although the effectiveness levels covered a wide range, from 24% (95% CI 10%–36%) to 72% (95% CI 46%–85%) (*9*–*15*).

We previously estimated the vaccine effectiveness (VE) of PPV23 for part of the vaccination program (June 15, 2020–September 18, 2021) (*9*). In this new study, we aimed to evaluate the real-life effectiveness of PPSV23 against IPD in persons >65 years of age in Denmark after a widely accepted vaccination program that ran during the period April 22, 2020–March 15, 2023. The longer study period and the inclusion of persons who turned 65 years of age during the study period enabled us to estimate VE for specific serotypes and for serotypes included in different types of pneumococcal vaccines, thereby adding new knowledge to the previous study. The primary outcomes were VE against all-type IPD and PPSV23-vaccine type IPD. Exploratory outcomes were VE against PPSV23-vaccine type IPD excluding serotype 3, VE against the most common IPD-causing serotypes in Denmark during the study period (3, 8, and 22F), and VE against serotypes present in PPSV23 but not in the now available 15-valent pneumococcal conjugate vaccine (PCV15) (i.e., serotypes 8, 10A, 11A, 12F, 15B, 2, 9N, 17F, and 20) or in the 20-valent pneumococcal conjugate vaccine (PVC20) (i.e., serotypes 2, 9N, 17F, and 20). In addition, we estimated VE over time, where statistical power allowed. The aim of this study was to explore the unique context of widespread PPSV23 vaccination directed at an entire population >65 years of age in a high-income country. We sought to gain insights into the effectiveness of PPSV23 vaccination 2 years after its introduction. The investigation includes extensive nationwide data within a high vaccination coverage setting, incorporating serotype-specific analyses.

## Methods

All residents of Denmark are assigned a unique personal identification number known as a CPR number. The CPR number enables person-level linkage among a variety of nationwide registries (*16*). This study used 4 registries: The Danish Civil Registration System (CPR register), The Danish Vaccination Register (DVR), the Danish Microbiology Database (MiBa), and the Danish National Patient Registry (DNPR).

We retrieved information on age, sex, and migration from the CPR register. Using Anatomic Therapeutic Chemical (ATC) classification codes (Appendix Table 1), we retrieved information on vaccine type and date of administration from the DVR, in which all administered vaccines have been recorded by law since 2015 (*17*). We obtained information on comorbidities within 5 years of study entry and exit from the DNPR, which holds information on all hospital admissions (except to psychiatric wards) with diagnoses coded according to the International Classification of Diseases, 10th Revision (ICD-10; Appendix Table 4) (*16*). We only considered the primary diagnosis. Selected comorbidities were based on the Charlson Comorbidity score (*18*,*19*) but counted as individual comorbidities and not given a score. We retrieved microbiological data from MiBa, which holds information on all microbiological test results in Denmark (*20*). In this study, an IPD case was defined as a positive diagnostic test for *S. pneumoniae* from cerebrospinal fluid, blood, or other normally sterile sites (e.g., pleura). All registries are updated regularly and contain near–real-time information.

The study period started on April 22, 2020, and ended on March 15, 2023. We chose the end date to allow time for potential cases of IPD to develop in persons vaccinated at the end of the program, and extracted data on IPD on March 29, 2023, to allow for a delay in registration. We collected data on previously administered vaccines for all persons in the cohort: influenza vaccine within 2 years before study entry, and PCV7 or PCV13 at any time before study entry. The data covering the period from June 15, 2020–September 18, 2021, was also presented in the previous study (*1*).

We included all residents in Denmark who were >65 years of age or turned 65 years of age during the study period. We excluded persons who had evidence of previous laboratory-confirmed IPD because they are at increased risk for another IPD episode (*21*,*22*). We also excluded persons who received a PPSV23 vaccine within 6 years before study entry so that we could assess VE for persons vaccinated within the vaccination program. Previous studies have shown a waning effect of PPSV23 VE over time (*23*–*26*), resulting in a recommendation of re-vaccination every 6th year in Denmark (*27*). We followed all persons in the study until the date of IPD diagnosis, emigration, death, or the end of the study period.

Because the immune response to vaccination is delayed by 2–3 weeks (*28*,*29*), persons were censored from the unvaccinated group at date of vaccination and entered the vaccinated group 14 days after vaccination; that is, they were censored from the study during the 14 days in between. Hence, persons accrued person-time in each group when appropriate. For the analyses regarding specific serotypes or groups of specific serotypes, persons were censored with an event at date of IPD if the disease was caused by the serotype(s) of interest, otherwise they were considered to be without an event.

We conducted 2 sensitivity analyses. The first analysis assessed whether there was any effect of censoring 0–14 days after vaccination. In the main analyses, participants were censored from day 0–14 after vaccination, and in the first sensitivity analysis, they were included as unvaccinated until 14 days after vaccination. The second analysis assessed whether there was any effect of including persons previously vaccinated with PCV. In the main analyses, participants were included regardless of previous vaccination with PCV. In the second sensitivity analysis, participants who had received PCV at any time before the study were excluded, and those who received PCV during the study period were censored at date of vaccination.

We calculated VE estimates by using a Cox regression model to estimate hazard ratios (HRs) with calendar time as the underlying timescale. We adjusted the estimates for age (restricted cubic spline), sex (male/female), and comorbidities (restricted cubic spline). We estimated VE using the formula 1 – HR × 100% with 95% CIs. We estimated waning VE by considering time intervals since vaccination: 14 days to 1 year, 1–2 years, and 2–3 years. Hence, some persons might contribute to risk time in all periods moving from group to group as time passes. We made all analyses by using R version 4.2.2 (The R Foundation for Statistical Computing, https://www.r-project.org).

## Results

During the study period, approximately 6,221,398 persons resided in Denmark; approximately 1,358,914 were >65 years of age or turned 65 years of age during the study period. We excluded 100,448 persons vaccinated with PPSV23 within 6 years before study entry, and 3,952 persons who had prior IPD. Because of censoring 14 days after vaccination, 16 persons vaccinated on April 22, 2020, who died within 14 days of vaccination, did not contribute person-time in the study. The final study cohort consisted of 1,254,498 persons (Figure), corresponding to 3,196,988 person-years of follow-up.

**Figure:**
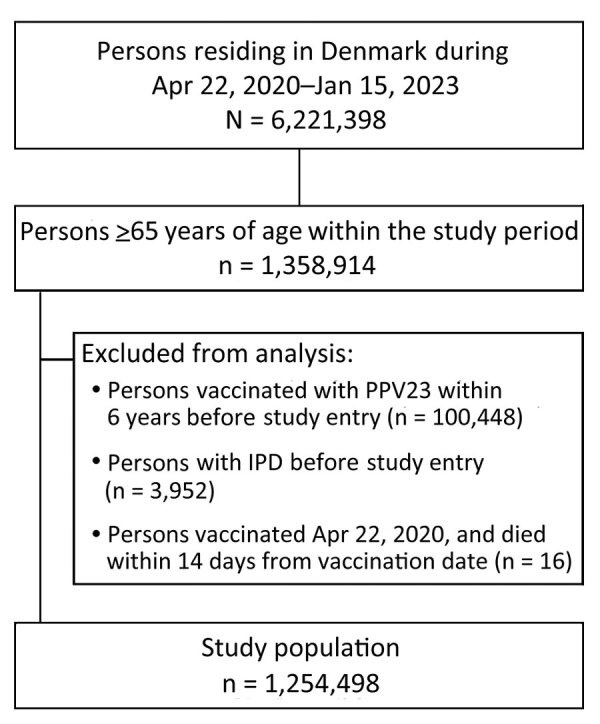
Flowchart of cohort development for follow-up study of effectiveness of 23-valent pneumococcal polysaccharide vaccine against invasive pneumococcal disease, Denmark.

We stratified the characteristics of included persons at entry date and at the end of follow-up by vaccination status (Table 1). The median age at entry was 72 years (interquartile range [IQR] 67–78 years), 74 years (IQR 68–81 years) for those unvaccinated at exit, and 75 years (IQR 70–81 years) for those vaccinated with PPSV23 at exit. Most (77%) of the persons in the study subjects were vaccinated with PPSV23 during the study period. Persons in the PPSV23 vaccinated group were more likely than those in the PPSV23 unvaccinated group to have been vaccinated against influenza within 2 years before study entry (55% vs. 21%) and during the study period (97% vs. 34%).

**Table 1:** Patient characteristics according to vaccination status in follow-up study of effectiveness of PPSV23 vaccine against invasive pneumococcal disease, Denmark*

Characteristic	Study population at entry	Study population at exit	
		Unvaccinated	Vaccinated with PPSV23 during the study period
Total	1,254,498 (100)	288,383	966,115
Sex			
F	668,726 (53)	157,590 (55)	511,136 (53)
M	585,772 (47)	130,793 (45)	454,979 (47)
Age, y			
Median (IQR)	72 (67–78)	74 (68–81)	75 (70–81)
<75	760,980 (61)	150,620 (52)	436,070 (45)
75–84	373,294 (30)	91,009 (32)	391,251 (41)
>85	120,224 (9)	46,754 (16)	138,794 (14)
Vaccination status			
Received PCV7 or PCV13 before study entry	19,035 (2)	2,494 (1)	16,541 (2)
Received PCV7 or PCV13 during follow-up	NA	1,500 (1)	15,558 (2)
Received influenza vaccine within 2 y before study entry	645,980 (52)	59,105 (21)	530,467 (55)
Received of influenza vaccine during follow-up	NA	97,400 (34)	937,489 (97)
No. underlying conditions†			
0	1,017,954 (81)	204,788 (71)	652,863 (68)
1	198,611 (16)	60,364 (21)	231,957 (24)
2	32,047 (3)	17,334 (6)	61,742 (6)
>3	5,886 (0)	5,897 (2)	19,553 (2)
Individual underlying conditions†			
Myocardial infarction	18,684 (1)	4,433 (2)	16,507 (2)
Congestive heart failure	15,256 (1)	7,624 (3)	26,295 (3)
Peripheral vascular disease	23,780 (2)	8,761 (3)	35,072 (4)
Cerebrovascular disease	50,748 (4)	16,028 (6)	55,803 (6)
Dementia	16,044 (1)	7,354 (3)	25,564 (3)
Chronic pulmonary disease	24,074 (2)	7,948 (3)	36,996 (4)
Connective tissue disease	10,765 (1)	4,955 (2)	23,406 (2)
Ulcer disease	8,893 (1)	2,693 (1)	8,755 (1)
Mild liver disease	3,512 (0)	2,277 (1)	6,328 (1)
Diabetes mellitus	15,130 (1)	3,699 (1)	14,362 (1)
Hemiplegia	621 (0)	347 (0)	1,407 (0)
Moderate/severe renal disease	11,104 (1)	6,404 (2)	20,725 (2)
Diabetes with chronic complications	7,301 (1)	4,806 (2)	16,603 (2)
Any tumor	67,451 (5)	29,878 (10)	109,912 (11)
Leukemia	715 (0)	1,087 (0)	4,157 (0)
Lymphoma	1,471 (0)	1,969 (1)	7,998 (1)
Moderate/severe liver disease	1,120 (0)	823 (0)	1,681 (0)
Metastatic solid tumor	4,785 (0)	3,271 (1)	7,573 (1)
AIDS	37 (0)	159 (0)	539 (0)

*Values are no. (%) except as indicated. Persons vaccinated with PPSV23 during follow-up contributed to the analysis for both unvaccinated and vaccinated. IQR, interquartile range; NA, not applicable; PCV7, 7-valent pneumococcal conjugate vaccine; PCV13, 13-valent pneumococcal conjugate vaccine; PPSV23, 23-valent polysaccharide pneumococcal vaccine. †Within 5 y before study entry (1st column) or within 5 y from end of follow-up (2nd and 3rd columns).

A total of 513 persons (200 in the unvaccinated group and 313 in the vaccinated group) had IPD caused by any serotype during the study period (Table 2). For PPSV23-serotype IPD, 134 events occurred in the unvaccinated group and 186 in the vaccinated group, whereas when serotype 3 was excluded, 105 events occurred in the unvaccinated group and 102 in the vaccinated group. For specific serotypes analyzed in the unvaccinated versus vaccinated groups, there were 29 versus 84 serotype 3 events, 32 versus 27 serotype 8 events, and 25 versus 9 serotype 22F events.

**Table 2:** Effectiveness of PPSV23 vaccine against IPD compared with no vaccination in follow-up study, Denmark, April 22, 2020–March 15, 2023*

IPD type	Vaccination status	No. events	Person-years	VE, % (95% CI)	
				Unadjusted	Adjusted†
All	Unvaccinated	200	1,277,147	Referent	
	Vaccinated	313	1,919,841	26 (11–39)	32 (18–44)
PPSV23 serotype	Unvaccinated	134	1,277,147	Referent	
	Vaccinated	186	1,919,841	36 (19–49)	41 (25–53)
PPSV23 serotype excluding serotype 3	Unvaccinated	105	1,277,147	Referent	
	Vaccinated	102	1,919,841	54 (38–65)	58 (43–68)
Serotype 3	Unvaccinated	29	1,277,147	Referent	
	Vaccinated	84	1,919,841	−25 (−95 to 20)	−17 (−83 to 25)
Serotype 8	Unvaccinated	32	1,277,147	Referent	
	Vaccinated	27	1,919,841	56 (27–74)	62 (36–77)
Serotype 22F	Unvaccinated	25	1,277,147	Referent	
	Vaccinated	9	1,919,841	86 (70–94)	88 (75–95)
PPSV23 non-PCV15 serotype‡	Unvaccinated	67	1,277,147	Referent	
	Vaccinated	71	1,919,841	45 (21–61)	50 (28–65)
PPSV23 non-PCV20 serotype§	Unvaccinated	18	1,277,147	Referent	
	Vaccinated	14	1,919,841	57 (2–81)	61 (10–83)

*IPD, invasive pneumococcal disease; PCV15, 15-valent pneumococcal conjugate vaccine; PCV20, 20-valent pneumococcal conjugate vaccine; PPSV23, 23-valent polysaccharide pneumococcal vaccine; VE, vaccine effectiveness. †Adjusted for age and comorbidities as a restricted cubic spline and sex as a categorical variable. ‡Serotypes 8, 10A, 11A, 12F, 15B, 2, 9N, 17F, and 20. §Serotypes 2, 9N, 17F, and 20.

The estimated VE against all-type IPD was 32% (95% CI 18%–44%), and VE against PPSV23-serotype IPD was 41% (95% CI 25%–53%) (Table 2). When estimating VE for PPSV23-serotype IPD but not considering serotype 3 as an event, we found a higher VE of 58% (95% CI 43%–68%). VE against serotype 3 was −17% (95% CI −83% to 25%). We found the highest VE estimates for serotype 8 (62% [95% CI 36%–77%]) and serotype 22F (88% [95% CI 75%–95%]). VE for PPSV23 non-PCV15 serotype IPD (8, 10A, 11A, 12F, 15B, 2, 9N, 17F, and 20) was 50% (95% CI 28%–65%). VE for PPSV23 non-PCV20 serotype IPD (2, 9N, 17F, and 20) was 61% (95% CI 10%–83%), largely powered by IPD caused by serotype 9N (21/32 events; data not shown). We were unable to assess VE against the PCV15/PCV20 non-PPSV23 serotype 6A because only 3 cases of IPD were caused by this serotype. 

The sensitivity analysis excluding persons who received PCV before study entry and censoring those who received PCV during the study period showed only minor changes in the point estimates compared with the estimates calculated when those persons were included. Most estimates increased slightly except against all-type IPD or IPD caused by serotype 22F (Appendix Table 2). The sensitivity analysis including persons as unvaccinated day 0–14 after vaccination showed no difference in the VE estimates compared with the estimates calculated when persons in that time period were excluded (Appendix Table 3).

When estimating VE over time (Table 3), we found that VE remained stable but declined slightly 2 years after vaccination. For all-type IPD, the VE was reduced from 39% to 27%. VE against PPSV23-serotype IPD declined from 52% to 43% and from 65% to 57% when excluding serotype 3. Protection against serotype 8 was high the first year after vaccination (86% [95% CI 54%–96%]), but estimates after 1–2 years were not significant (46% [95% CI −35% to 80%]). VE against serotype 22F remained very high, at 92% to 86%, but few events occurred (*34*).

**Table 3:** Hazard ratios and effectiveness of PPSV23 vaccine against IPD in follow-up study, comparing time since vaccination with PPSV23 with no vaccination, Denmark, April 22, 2020–March 15, 2023*

IPD type	Vaccination status	No. events	Person-years	VE, % (95% CI)	
				Unadjusted	Adjusted†
All	Unvaccinated	200	1,277,147	Referent	
	0–1 y after vaccination	74	838,041	38 (18–53)	39 (19–53)
	1–2 y after vaccination	131	728,480	24 (3–40)	30 (10–45)
	2–3 y after vaccination	108	353,319	14 (−15 to 36)	27 (2–46)
PPSV23 serotype	Unvaccinated	134	1,277,147	Referent	
	0–1 y after vaccination	38	838,041	51 (29–66)	52 (30–67)
	1–2 y after vaccination	85	728,480	25 (−2 to 44)	30 (5–48)
	2–3 y after vaccination	63	353,319	34 (5–54)	43 (19–61)
PPSV23 serotype excluding serotype 3	Unvaccinated	105	1,277,147	Referent	
	0–1 y after vaccination	23	838,041	64 (43–78)	65 (44–78)
	1–2 y after vaccination	44	728,480	47 (22–64)	52 (29–67)
	2–3 y after vaccination	35	353,319	49 (20–68)	57 (32–73)
Serotype 3	Unvaccinated	29	1,277,147	Referent	
	0–1 y after vaccination	15	838,041	−2 (−95 to 46)	−2 (−94 to 47)
	1–2 y after vaccination	41	728,480	−47 (−148 to 13)	−39 (−135 to 18)
	2–3 y after vaccination	28	353,319	−14 (−110 to 38)	−1 (−87 to 46)
Serotype 8	Unvaccinated	32	1,277,147	Referent	
	0–1 y after vaccination	3	838,041	86 (52–96)	86 (54–96)
	1–2 y after vaccination	15	728,480	37 (−24 to 68)	46 (−8 to 73)
	2–3 y after vaccination	9	353,319	34 (−71 to 74)	48 (−35 to 80)
Serotype 22F	Unvaccinated	25	1,277,147	Referent	
	0–1 y after vaccination	1	838,041	92 (40–99)	92 (39–99)
	1–2 y after vaccination	3	728,480	88 (59–96)	89 (64–97)
	2–3 y after vaccination	5	353,319	82 (47–94)	86 (60–95)

*IPD, invasive pneumococcal disease; PCV15, 15-valent pneumococcal conjugate vaccine; PCV20, 20-valent pneumococcal conjugate vaccine; PPSV23, 23-valent polysaccharide pneumococcal vaccine; VE, vaccine effectiveness. †Adjusted for age and comorbidities as a restricted cubic spline and sex as a categorical variable.

## Discussion

Using nationwide data, this cohort study shows that PPSV23 vaccination in persons >65 years old is associated with protection against all-type IPD, IPD caused by PPSV23 vaccine serotypes, and IPD caused by serotypes 8 and 22F, specifically, but not IPD caused by serotype 3. Studies from Spain, Israel, Taiwan, England, and Wales, as well as our previous study from Denmark, have shown PPSV23 VE against all-type IPD ranging from 42% (95% CI 19%–59%) to 70% (95% CI 48%–82%) (*1*,*10*,*30*–*32*) and against PPSV23-serotype IPD ranging from 24% (95% CI 10%–36%) to 72% (95% CI 46%–85%) in persons >60 years of age (*1*,*10*–*15*). In this study, we found a lower estimate against all-type IPD (VE 32%), which could be the result of the higher age of our study population because the effect of PPSV23 lessens with age (*33*). The difference could also be because of differences in serotype distribution or comorbidities in the study populations, but unfortunately, not all studies have provided this level of detail or include the same comorbidities. Our estimate against PPSV23-serotype IPD (VE 41%) falls within the range of the VE found in previous studies. Compared with our previous study on PPSV23 in Denmark (*1*), this follow-up study finds a lower VE against all-type IPD (42% vs. 32%) and PPSV23-vaccine type IPD (58% vs. 41%). This study covers the entire vaccination program and includes persons offered PPSV23 at the beginning of the program; that is, more vulnerable groups where the vaccine might not have the same effect. In this study, persons who received a vaccination were censored for 14 days after vaccination. In contrast, they were included in the unvaccinated group in the previous study. In this study, we performed a sensitivity analysis investigating the effect of censoring persons 0–14 days after vaccination, which did not affect the VE estimates (Appendix Table 3).

Previous research has shown that the VE against IPD caused by serotype 3 is low to nonexistent, whereas the VE against IPD caused by serotype 8 or 22F varies from low to moderate (*11*,*13*,*34*–*36*). Our study also showed no effect against IPD caused by serotype 3; therefore, we estimated VE against PPSV23-vaccine type IPD excluding serotype 3. As expected, and seen before for overall VE against IPD (*34*), this estimate yields an increased VE of 58%, compared with 41%, and indicates that a VE including all PPSV23 vaccine serotypes might underestimate the effectiveness of the vaccine against the other 22 serotypes because of the poor effect against serotype 3. Because serotype 3 continues to cause the most IPD cases of any serotype in those >65 years of age in Denmark, comparing studies including all PPSV23 vaccine serotypes shows the effectiveness of the vaccine on all the IPD cases that could potentially be prevented by PPSV23. However, that the effectiveness against serotype 3 clearly is very low. Estimating VE against PPSV23-serotype IPD excluding serotype 3 provides a better idea of how the vaccine works against the rest of the serotypes grouped together and of how many cases we can actually hope to prevent by using this vaccine.

In Denmark, serotype replacement is taking place, similar to what has been seen in other countries, and policy makers have to consider the indirect effect of pediatric vaccination against pneumococcal disease on the adult population (*37*). The incidence of IPD caused by serotype 8 increased after the introduction of PCVs into the childhood vaccination program (*38*,*39*); those vaccines do not include serotype 8 (*40*), leaving room for that serotype to advance. In Denmark, such an increase occurred in age groups >15 years, and especially for persons >65 years of age (*38*). Our study shows that PPSV23 is effective against serotypes 8 and 22F and that a vaccination program might be an important factor in protecting persons >65 years of age against IPD caused by those serotypes. On the other hand, PCV13 vaccination in children in Denmark has not reduced the incidence of serotype 3 (*41*) in the population, and in our study, we still see serotype 3 causing most IPD cases among older adults. Unfortunately, vaccination with PPSV23 is unlikely to reduce this burden because of the lack of effect on IPD caused by serotype 3. Other conjugated vaccines, such as PCV15 and PCV20, offer a broader serotype coverage than PCV13. Both contain 1 serotype that is not included in PPSV23 (serotype 6A) (*42*), whereas PPSV23 contains 9 serotypes not included in PCV15 and 4 serotypes not included in PCV20 (*28*). Of 513 cases of IPD included in this study, 138 cases were caused by the PPSV23 non-PCV15 serotypes, 32 by the PPSV23 non-PCV20 serotypes, and 3 by the PCV15/PCV20 non-PPSV23 serotype 6A. Our findings indicate that persons >65 of age still suffer from IPD caused by PPSV23 non-PCV15/PCV20 serotypes, and that PPSV23 offers protection, having a VE of 50% for PPSV23 non-PCV15 serotypes and 61% for PPSV23 non-PCV20 serotypes.

The first limitation of this study is that, for some analyses, few events occurred, which decreases the statistical power and results in wide 95% CIs when estimating VE. That finding was especially true for the PPSV23 non-PCV20 estimate. In this study, we also did not have enough follow-up time to evaluate a waning effect beyond 3 years. However, we can conclude that only minor waning was seen during the study period. To detect the long-term durability of protection, a follow-up study should be conducted.

Healthy vaccinee bias cannot be ruled out. In this study, 21% of unvaccinated persons received influenza vaccination before the study, compared with 55% among the vaccinated persons (Table 1). That finding indicates that persons who accept 1 vaccination are more likely to accept another and that those vaccinated in general are more adherent to the recommendations from public health authorities. However, the vaccinated persons are approximately the same age and have the same distribution of comorbidities as the unvaccinated persons, which speaks against a healthy vaccinee bias. The similar distribution of comorbidities between the groups speaks against the opposite bias, confounding by indication, where those who are more at risk might be more likely to get vaccinated. To account for both those biases, we adjusted our results for comorbidities. Another limitation to this study is that the difference in influenza vaccination coverage between the groups of PPSV23 vaccinated (97%) and nonvaccinated (34%) persons increased during the study period because influenza vaccination might also reduce the risk for IPD (*43*), which could lead to an overestimation of the PPSV23 VE.

This study is strengthened by the comprehensiveness of the Denmark registries, enabling inclusion of all residents >65 years of age. Similar to our previous study on PPSV23 VE in Denmark (*1*), the study period spans a time in which nonpharmacologic restrictions were in place due to COVID-19. However, our study covers a longer period after those restrictions were lifted, when Denmark experienced a return to more normal levels of IPD cases. Thus, we believe that the VE found in this study reflects the VE in a setting without extraordinary restrictions.

In conclusion, this study shows that persons >65 years of age who are vaccinated with PPSV23 are protected against all-type IPD, PPSV23-serotype IPD, IPD caused by serotypes 8 and 22F, and IPD caused by PPSV23 non-PCV15/PCV20 serotypes, but not against IPD caused by serotype 3. In addition, the protection persists; VE only wanes marginally and insignificantly over almost 3 years. These findings support a vaccination program with PPSV23 against IPD to protect those >65 years of age.

Appendix Additional information for follow-up study of effectiveness of 23-valent pneumococcal polysaccharide vaccine against invasive pneumococcal disease, Denmark.
