# Supplementary material for: Follow-Up Study of Effectiveness of 23-Valent Pneumococcal Polysaccharide Vaccine Against All-Type and Serotype-Specific Invasive Pneumococcal Disease, Denmark
Source: Emerg Infect Dis. 2024 Jun;30(6):1164–72. doi: 10.3201/eid3006.230975 (PMC11138992; doi:10.3201/eid3006.230975)
Supplement: Appendix — Additional information for follow-up study of effectiveness of 23-valent pneumococcal polysaccharide vaccine against invasive pneumococcal disease, Denmark. [file 23-0975-Techapp-s1.pdf]

Article DOI: https://doi.org/10.3201/eid3006.230975
EID cannot ensure accessibility for supplementary materials supplied by authors. Readers who have difficulty accessing supplementary content should contact the authors for assistance.

Follow-Up Study of Effectiveness of 23-Valent Pneumococcal Polysaccharide Vaccine Against All-Type and Serotype-Specific Invasive Pneumococcal Disease, Denmark

Appendix

Appendix Table 1. ATC codes used for data extraction*

Vaccine type	ATC code
PCV7	J07AL02
PCV13	J07AL02
PPSV23	J07AL01
Influenza	J07BB01, J07BB02

*ATC, anatomic therapeutic chemical; PCV7, 7-valent pneumococcal conjugate vaccine; PCV13, 13-valent pneumococcal conjugate vaccine; PPSV23, 23-valent polysaccharide pneumococcal vaccine.

Appendix Table 2. Sensitivity analysis of vaccine effectiveness against all-type IPD, PPSV23-serotype IPD, PPSV23-serotype IPD without serotype 3, PPSV23 non-PCV15 serotypes, PPSV23 non-PCV20 serotypes, serotype 3 IPD, serotype 8 IPD and serotype 22F IPD comparing vaccination with PPSV23 with no vaccination, April 22, 2020 to March 15, 2023 excluding those with PCV-vaccination before study entry and censoring those with PCV-vaccination during follow-up*

				VE, % (95% CI)	
Outcomes	Vaccination status	No. events	PYRS	Unadjusted	Adjusted†
All-serotype IPD	Unvaccinated	192	1,257,958	Referent	
	Vaccinated	295	1,853,321	26 (10–39)	32 (17–44)
PPSV23-serotype IPD	Unvaccinated	131	1,257,958	Referent	
	Vaccinated	175	1,853,321	37 (19–50)	41 (26–54)
PPSV23-serotype IPD (excluding serotype 3)	Unvaccinated	102	1,257,958	Referent	
	Vaccinated	94	1,853,321	55 (39–66)	59 (44–69)
Serotype 3 IPD	Unvaccinated	29	1,257,958	Referent	
	Vaccinated	81	1,853,321	−23 (−93 to 21)	−15 (−81 to 26)
Serotype 8 IPD	Unvaccinated	32	1,257,958	Referent	
	Vaccinated	25	1,853,321	59 (31–76)	64 (39–79)
Serotype 22F IPD	Unvaccinated	23	1,257,958	Referent	
	Vaccinated	9	1,853,321	85 (67–93)	87 (72–94)
PPSV23 non-PCV15 serotypes IPD (8, 10A, 11A, 12F, 15B, 2, 9N, 17F and 20)	Unvaccinated	67	1,257,958	Referent	
	Vaccinated	66	1,853,321	48 (25–64)	52 (32–67)
PPSV23 non-PCV20 serotypes IPD (2, 9N, 17F and 20)	Unvaccinated	18	1,257,958	Referent	
	Vaccinated	13	1,853,321	60 (8–82)	64 (15–85)

*CI, confidence interval; IPD, invasive pneumococcal disease; PCV15, 15-valent pneumococcal conjugate vaccine; PCV20, 20-valent pneumococcal conjugate vaccine; PPSV23, 23-valent polysaccharide pneumococcal vaccine; PYRS, person years; VE, vaccine effectiveness.
†Adjusted for age and comorbidities as a restricted cubic spline and sex as a categorical variable.

Appendix Table 3. Sensitivity analysis of vaccine effectiveness against all-type IPD, PPSV23-serotype IPD, PPSV23-serotype IPD without serotype 3, PPSV23 non-PCV15 serotypes, PPSV23 non-PCV20 serotypes, serotype 3 IPD, serotype 8 IPD and serotype 22F IPD comparing vaccination with PPSV23 with no vaccination, April 22, 2020 to March 15, 2023 including those 0–14 d after vaccination in the unvaccinated group*

				VE, % (95% CI)	
Outcomes	Vaccination status	No. events	PYRS	Unadjusted	Adjusted†
All-serotype IPD	Unvaccinated	204	1,314,165	Referent	
	Vaccinated	313	1,919,841	26 (11–39)	32 (18–44)
PPSV23-serotype IPD	Unvaccinated	135	1,314,165	Referent	
	Vaccinated	186	1,919,841	35 (18–49)	40 (24–53)
PPSV23-serotype IPD (without serotype 3)	Unvaccinated	106	1,314,165	Referent	
	Vaccinated	102	1,919,841	53 (38–65)	57 (43–68)
Serotype 3 IPD	Unvaccinated	29	1,314,165	Referent	
	Vaccinated	84	1,919,841	−29 (−100 to 17)	−21 (−88 to 23)
Serotype 8 IPD	Unvaccinated	32	1,314,165	Referent	
	Vaccinated	27	1,919,841	56 (24–74)	61 (33–78)
Serotype 22F IPD	Unvaccinated	25	1,314,165	Referent	
	Vaccinated	9	1,919,841	86 (69–94)	88 (74–95)
PPSV23 non-PCV15 serotypes IPD (8, 10A, 11A, 12F, 15B, 2, 9N, 17F and 20)	Unvaccinated	68	1,314,165	Referent	
	Vaccinated	71	1,919,841	45 (22–61)	50 (28–65)
PPSV23 non-PCV20 serotypes IPD (2, 9N, 17F and 20)	Unvaccinated	19	1,314,165	Referent	
	Vaccinated	14	1,919,841	58 (6–81)	63 (14–84)

*CI, confidence interval; IPD, invasive pneumococcal disease; PCV15, 15-valent pneumococcal conjugate vaccine; PCV20, 20-valent pneumococcal conjugate vaccine; PPSV23, 23-valent polysaccharide pneumococcal vaccine; PYRS, person years; VE, vaccine effectiveness.
†Adjusted for age and comorbidities as a restricted cubic spline and sex as a categorical variable.

Appendix Table 4. ICD-10 codes for comorbidities.

Individual comorbidities	ICD-10 codes
Myocardial infarction	DI21, DI22, DI23
Congestive heart failure	DI50, DI110, DI130, DI132
Peripheral vascular disease	DI70, DI71, DI72, DI73, DI74, DI77
Cerebrovascular disease	DI60, DI70, DG45, DG46
Dementia	DF00, DF04, DF051, DG30
Chronic pulmonary disease	DJ40, DJ48, DJ60, DJ68, DJ684, DJ701, DJ703, DJ841, DJ920, DJ961, DJ982, DJ983
Connective tissue disease	DM30, DM37, DM05, DM06, DM08, DM09, DD86
Ulcer disease	DK221, DK25, DK29
Mild liver disease	DK700, DK704, DB18, DK709, DK71, DK73, DK74, DK760
Diabetes mellitus	DE100, DE101, DE109, DE110, DE111, DE119
Hemiplegia	DG81, DG82
Moderate/severe renal disease	DN00, DN06, DN17, DN20, DQ61, DI12, DI13, DN07, DN11, DN14
Diabetes with chronic complications	DE102, DE109
Any tumor	DC00, DC76
Leukemia	DC91, DC96
Lymphoma	DC81, DC86, DC88, DC90, DC96
Moderate/severe liver disease	DB150, DB160, DB162, DB190, DK704, DK72, DK766, DI85
Metastatic solid tumor	DC76, DC81
AIDS	DB21, DB25
